# Integrated multi‐omics data reveals the molecular subtypes and guides the androgen receptor signalling inhibitor treatment of prostate cancer

**DOI:** 10.1002/ctm2.655

**Published:** 2021-12-22

**Authors:** Jialin Meng, Xiaofan Lu, Chen Jin, Yujie Zhou, Qintao Ge, Jun Zhou, Zongyao Hao, Fangrong Yan, Meng Zhang, Chaozhao Liang

**Affiliations:** ^1^ Department of Urology The First Affiliated Hospital of Anhui Medical University Hefei P. R. China; ^2^ Anhui Province Key Laboratory of Genitourinary Diseases Anhui Medical University Hefei P. R. China; ^3^ Institute of Urology Anhui Medical University Hefei P. R. China; ^4^ State Key Laboratory of Natural Medicines Research Center of Biostatistics and Computational Pharmacy China Pharmaceutical University Nanjing P. R. China; ^5^ Division of Gastroenterology and Hepatology Key Laboratory of Gastroenterology and Hepatology Ministry of Health Renji Hospital School of Medicine Shanghai Jiao Tong University Shanghai P. R. China; ^6^ Urology Institute of Shenzhen University the Third Affiliated Hospital of Shenzhen University Shenzhen University Shenzhen P. R. China

**Keywords:** androgen receptor signalling inhibitor, prostate cancer, molecular subtype, recurrence‐free survival

Dear editor,

Prostate cancer (PCa) is the most frequent malignant tumour in males,^1^ it is essential to precisely identify the specific molecular features and judge potential clinical outcomes from the multi‐omics aspect. Recently, we developed an R package “*MOVICS*” (https://xlucpu.github.io/MOVICS/MOVICS‐VIGNETTE.html) for multi‐omics integration and clustering, aim to stratify tumour molecular subtypes and facilitate precision medicine.^2^ In the current study, we firstly proposed the PCa multi‐omics classification (PMOC) system derived from mRNA, microRNA, long noncoding RNA, DNA methylation, and somatic mutation, using 10 leading‐edge clustering algorithms. We collected a total of 1192 PCa patients from five independent cohorts and an external AHMU‐PC cohort from our own institute^3^ (Tables S1 and S2). The technical details are listed in Supporting Information.

We identified three clusters independently from ten multi‐omics integrative clustering algorithms (Figure 1A) referring to the clustering prediction index, Gaps‐statistics analysis (Figure S1) and predefined PAM50 system,^4^ and further combined the clustering results via a consensus ensemble approach (Figure 1B). Multi‐omics data in PMOCs was visualized in Figure 1C. Significantly, diverse clinical recurrence‐free survival (RFS) outcomes were observed (all *p* < .001, Figure 1D). Most PMOC2 patients had a higher Gleason score than PMOC1 and PMOC3 (61.8% vs. 23.0% vs. 9.7%, *p* = .012), as well as the proportion of advanced pathology T stage (86.8% vs. 54.9% vs. 52.6%, *p* < 0.001, Table S3). The top 100 subtype‐specific markers for each PMOC were selected for the reproduction of PMOCs in external validation cohorts (Table S4).

**FIGURE 1:**
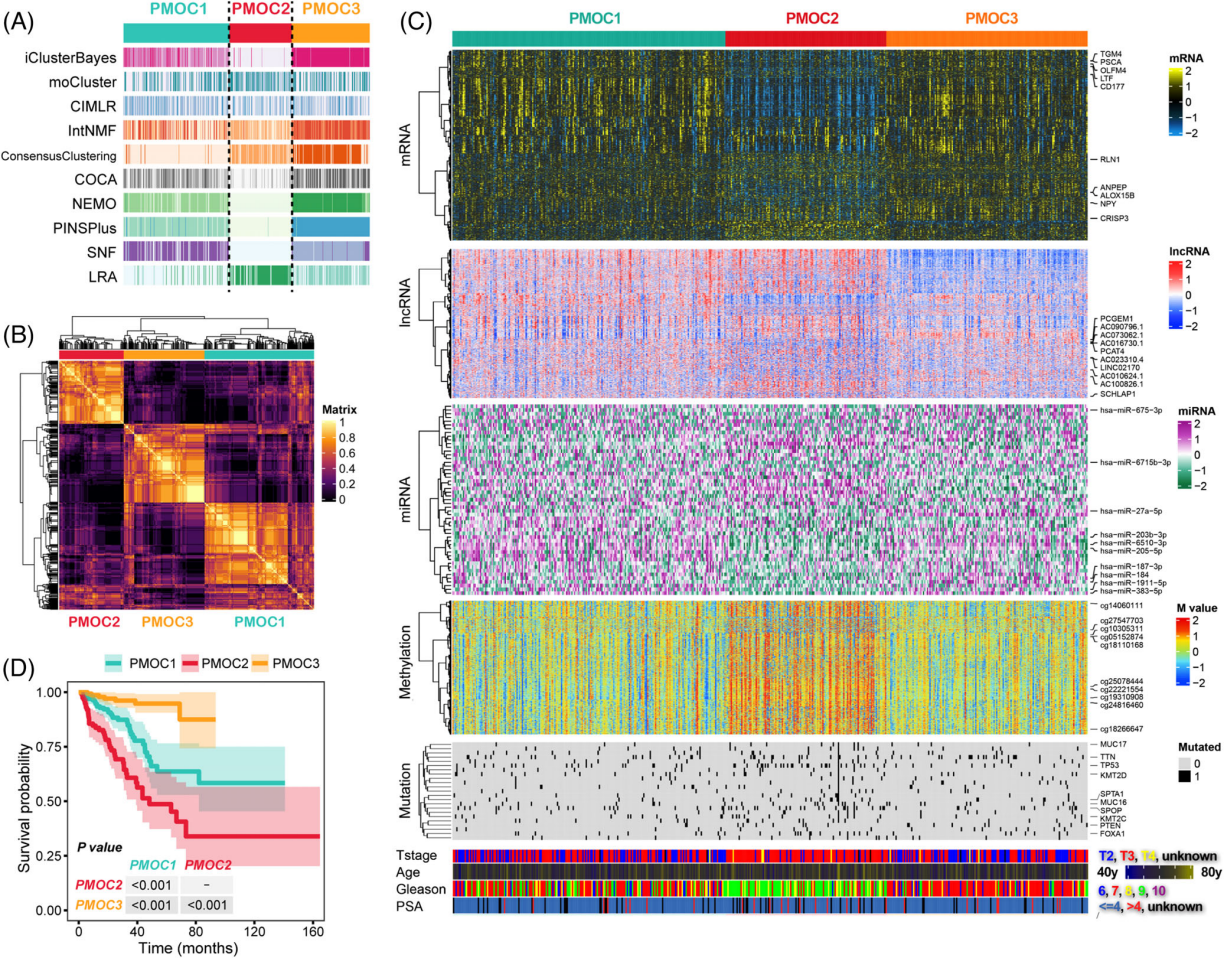
Recognition of the prostate cancer multi‐omics classification (PMOC) system in the TCGA‐PRAD cohort. (A) Clustering of prostate cancer (PCa) patients via 10 leading‐edge clustering methods. (B) Consensus matrix for three clusters based on the 10 algorithms. (C) Visualization of multi‐omics data for 1526 mRNAs, 242 lncRNAs, 30 miRNAs, 1073 DNA CpG methylation sites and 23 mutant genes. (D) Differential recurrence‐free survival outcome in three PMOCs, log‐rank test

We observed the significant activation of the G2M checkpoint, E2F target pathways in PMOC2 (Figure 2A). Specifically, the decreased phosphorylated protein levels of p‐CHK1 and p‐CHK2 in PMOC2 may weaken the inhibitory function of CDC25 components and increase CDK1 activation (Figure 2B). For PMOC1, we observed the activation of TNF‐α signalling, IL6/JAK/STAT3 and IL2/STAT5 signalling, which were immune response relevant. PMOC3 presented activation of both immune‐associated and oncogenic pathways; the phosphorylated mTOR and mTOR levels were activated and could further promote cell growth (Figure 2C).

**FIGURE 2:**
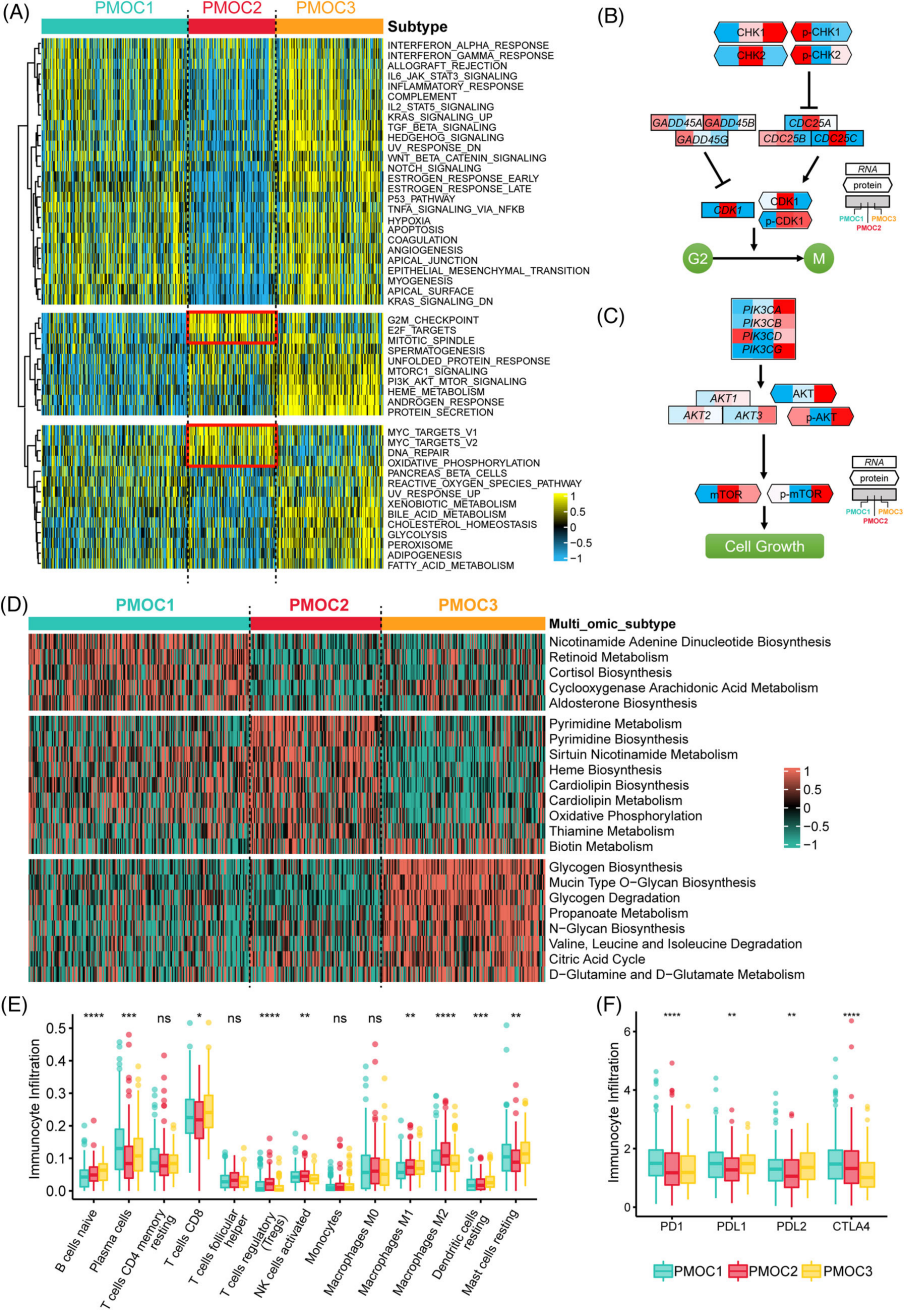
Differential activity of tumour‐associated pathways across three prostate cancer multi‐omics classifications (PMOCs). (A) Heatmap of 50 differentially activated HALLMARK pathways. (B) G2M pathways activated in the PMOC2 at both the mRNA and protein levels. (C) PI3K/AKT pathways activated in the PMOC3 at both the mRNA and protein levels. (D) Heatmap of subtype‐specific metabolism signalling pathways. (E) Differential infiltration of 13 immunocytes among three subtypes, Kruskal‐Wallis test. (F) Expression patterns of four immune checkpoints across three PCa subtypes, Kruskal‐Wallis test. ns, not significant; *p < .05; **p < .01; ***p < .001; ****p < .0001.

We further compared the activation status of metabolic pathways. The nicotinamide adenine dinucleotide biosynthesis and cyclooxygenase arachidonic acid metabolism pathways were activated in PMOC1, which were reported to be associated with tumour inflammation and immune‐metabolic circuits.^5^ In PMOC2, we observed the activated pyrimidine metabolism and biosynthesis, biotin metabolism, and oxidative phosphorylation. Glycogen metabolism and amino acid metabolism‐associated pathways were highly activated in PMOC3 (Figure 2D). PMOC3 had higher infiltration of immune‐suppressed components, while PMOC1 tended to exhibit immune activated components, and higher expression of PD1, PDL1 and CTLA4 (Figure 2E,F), and was also associated with immune activated molecular subtype^3^ (Figure S2A).

Genetic alteration contributed dramatically to shaping the subtypes. Specifically, the total tumour mutant burden was highest in PMOC2 (*p* < .001, Figure S3A). PMOC2 contained most patients with TP53 mutation (PMOC2: 23.6%, PMOC1: 8.6%, PMOC3: 5.8%, *p* < .001), and SPOP (PMOC2: 18.7%, PMOC1: 9.6%, PMOC3: 7.8%, *p* = .0138, Figure S3B, Table S5). The tumour suppressor APC protein is an antagonist of the Wnt signalling pathway.^6^ Mutant APC resulted in lower expression (*p* = .0026, Figure S3C) and led to an unfavourable clinical outcome (*p* = .013, Figure S3D). Both the lost and gained copy numbers were significantly increased in PMOC2 (Figure S4A). Interestingly, we observed that the gained copy number in PMOC2 was mostly located at the 8q24.21 region which was not amplified in either PMOC1 or PMOC3 (Figure S4B). The amplification and gain alteration of PVT1, an important gene located in the 8q24.21 region,^7^ occurred mostly in PMOC2 patients (*p* < .001, Figure S5A), PVT1 expression positively linked with its copy number alteration (*p* < .001, Figure S5B), and patients carried with PVT1 amplification suffered from the worst RFS (*p* = .003, Figure S5C).

In the PAM50 system, LumB has the worst prognosis (Figure S6A). PMOC2 remarkably overlapped with LumB and demonstrates the worst outcome. LumB/PMOC2 patients had an unfavourable outcome compared with LumB/PMOC1+PMOC3 patients (Figure S6B), which may be affected by DNA repair and replication pathways (Figure S6C), LumB/PMOC2 also had higher infiltration of anti‐inflammatory immunocytes (Figure S6D). TCGA research network reported a classifier of seven genetically distinct subtypes via the differential ERG/ETV1/ETV4/FLI1 fusion, or SPOP, FOXA1, IDH1 mutations.^8^ Of note, the PMOC system offered additional prognostic value to the existing classification scheme (Figure S2B,C).

Transcriptome regulatory networks play important role in the genesis and progression of tumours, we, therefore, evaluated the activity of 23 regulons and 71 chromatin remodelling regulons.^9^ Patients with PMOC2 are likely regulated by the human Fox gene family, patients in the PMOC3 group attracted us by the activation of androgen receptor (AR), epidermal growth factor receptor (EGFR), and hypoxia inducible factor 1 subunit alpha (HIF1A) (Figure 3A). PMOC3 significantly enriched the AR‐A score (*p *< .001, Figure 3B) and AR activation signature (*p* < .001, Figure 3C). In the androgen deprivation therapy (ADT)‐treated Abida cohort,^10^ PMOC2 demonstrated the activation of cell cycle‐associated pathways, and PMOC3 was linked with the activated androgen, estrogen and PI3K/AKT signalling pathways (Figure 3D). PMOC3 was more likely to respond to ADT therapy (response rate of 26.7% in PMOC3, Figure 3E), including bicalutamide (*p* = .089, Figure 3F). To further validate the differential activity of AR signalling, we represented the PMOC groups in the AHMU‐PC cohort (Figure 3G) by nearest template prediction (NTP) using subtype‐specific genes; we revealed that patients in PMOC3 from the AHMU‐PC cohort contained the highest activation of AR signalling (*p* < .001, Figure 3H), and more sensitive to bicalutamide (*p* = .0036, Figure 3I). In‐dept validations are warranted for the tight association between PMOC3 and anti‐androgen therapy. In addition, we represented the separation of PMOCs in external independent cohorts (Figure S7). In GSE54460, GSE70770, MSKCC and GSE116918 cohorts, all patients belonging to PMOC2 presented the worst clinical outcomes (all *p* < .05, Figure 3J). Moreover, our PMOC system remained the independent prognostic factor after adjusting other major clinical features in all five PCa patient cohorts (all *p* < .05, Table 1).

**FIGURE 3:**
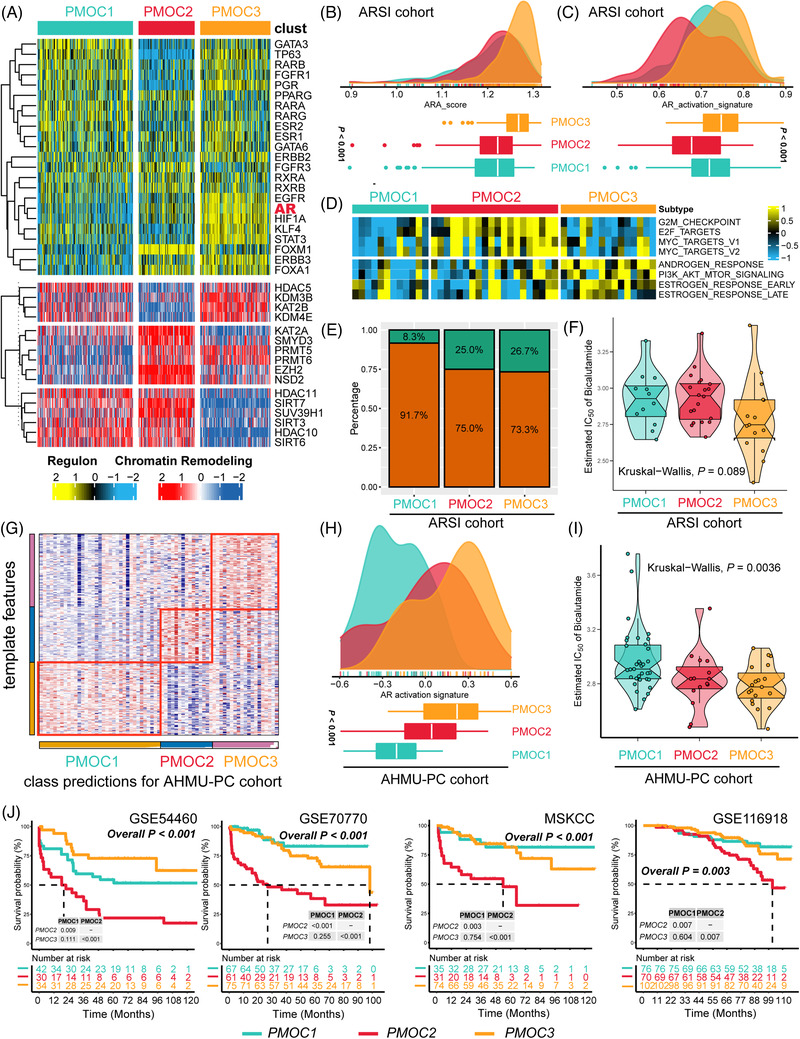
Differential response to ADT therapy across prostate cancer (PCa) genomic subtypes. (A) Activity status of 23 transcription factors and chromatin remodelling regulators. (B) Activation of the AR signalling pathway evaluated by AR‐A score and (C) AR activation signature among three prostate cancer multi‐omics classification (PMOC) subtypes in TCGA‐PRAD cohort, Kruskal‐Wallis test. (D) Differentially activated specific pathways in PMOC2 and PMOC3 validated in patients in the Abida cohort. (E) Patients in the PMOC3 subtype benefit more from ADT therapy. (F) Patients in the PMOC3 subtype were more suitable for bicalutamide treatment. (G) Representing the PMOCs in the AHMU‐PC cohort. (H) Distribution of AR activation signature among three PMOCs in AHMU‐PC cohort, Kruskal‐Wallis test; (I) Patients in the AHMU‐PC PMOC3 subtype were more suitable for bicalutamide treatment, Kruskal‐Wallis test. (J) Differential recurrence‐free survival outcome in reproduced PMOCs of external cohorts, log‐rank test

**TABLE 1:** Prostate cancer multi‐omics classifications (PMOCs) prognostic value after adjusting major clinicopathological features

	TCGA			GSE54460^$^			GSE116918			GSE70770^#^			MSKCC		
	HR	95% CI	*p*	HR	95% CI	*p*	HR	95% CI	*p*	HR	95% CI	*p*	HR	95% CI	*p*
Age	1.013	.981–1.047	.426	.999	.953–1.047	.957	.963	.923–1.005	.085	–	–	–	.988	.936–1.043	.667
PSA, > 4 versus < = 4 ng/dl	1.929	.901–4.127	.091	1.688	.379–7.526	.493	–	–	–	.702	.420–1.173	.177	.744	.286–1.939	.545
**Tumour stage**															
T1				ref.			ref.								
T2	ref.			.486	.210–1.121	.090	1.170	.441–3.107	.753	ref.			ref.		
T3	1.836	.964–3.496	.064	.548	.192–1.562	.260	1.900	.734–4.916	.186	1.802	.982–3.307	.057	2.943	1.291–6.712	.010*
T4	1.589	.340–7.415	.556				8.217	1.675–40.316	.009*	4.732	.915–24.472	.064	3.426	.902–13.005	.070
**Gleason**															
6	ref.			ref.			ref.			ref.			ref.		
7	3.812	.511–28.441	.192	2.885	.839–9.918	.093	2.141	.784–5.843	.137	6.299	1.445–27.451	.014*	3.602	.812–15.966	.092
8	5.134	.651–40.494	.121	6.461	1.410–29.601	.016*	1.910	.635–5.744	.249	9.236	1.721–49.567	.010*	23.239	4.392–122.957	<.001*
9	8.662	1.145–65.556	.037	3.426	.710–16.532	.125	1.279	.417–3.924	.667	31.970	6.340–161.172	<.001*	12.992	2.283–73.923	.004*
**PMOC**															
PMOC3	ref.			ref.			ref.			ref.			ref.		
PMOC2	5.593	2.373–13.182	.001*	.354	1.492–9.523	.005*	2.335	1.165–4.680	.017*	2.319	1.296–4.149	.005*	2.416	1.014–5.756	.047*
PMOC1	4.044	1.770–9.238	<.001*	.833	.449–2.765	.816	.939	.433–2.036	.874	.671	.289–1.559	.354	1.311	.453–3.792	.618

Abbreviations: CI, confidence interval; HR, hazard ratio, PMOC, prostate cancer multi‐omics classification.

^$^ T3 group contains T3 and T4, due to the small number of T4.

^#^ Gleason scores 5 + 6 as the reference in GSE70770.

Tumour biological process is complex with the internal cross‐talking between regulatory features from a different level, thus comprehensive data mining through multi‐omics profile is essential to decipher the tumour heterogeneity. We harnessed ten algorithms to recognize the PMOC system by the consensus clustering, more algorithms included, more stable and convincing the system is. Taken together, the PMOC1 “tumour‐inflammatory” subtype involves the activation of inflammation‐associated metabolism pathways and a high level of immune checkpoint proteins. The PMOC2 “tumour‐activated” subtype contains activated cell cycle and DNA repair pathways, a high rate of gene mutation, and 8q24.21 copy number amplification associated with poor prognosis. The PMOC3 “tumour‐balanced” subtype represents the activation of both oncogenic and proinflammatory pathways, links with a favourable prognosis, the enrichment of the AR response resulted in the suitability of ARSI treatment. This multi‐omics consensus PMOC system can further assist the precise and targeted clinical therapy for PCa patients.

## CONFLICT OF INTEREST

The authors declare that they have no conflict of interest.

## FUNDING INFORMATION

This work was supported by the National Natural Science Foundation of China (81630019, 81870519, 81802827 and 81973145), supporting project for Distinguished Young Scholar of Anhui Colleges (gxyqZD2019018), the National Key R&D Program of China (2019YFC1711000), the Key R&D Program of Jiangsu Province [Social Development] (BE2020694), the Scientific Research Foundation of the Institute for Translational Medicine of Anhui Province (2017ZHYX02), the Natural Science Foundation of Guangdong Province, China (2017A030313800), the Key project of provincial natural science research project of Anhui Colleges (KJ2019A0278), and 2017 Anhui Province special program for guiding local science and technology development by the central government (2017070802D148).

## Supporting information

Supporting Information
