# Supplementary material for: Integrated multi‐omics data reveals the molecular subtypes and guides the androgen receptor signalling inhibitor treatment of prostate cancer
Source: Clin Transl Med. 2021 Dec 22;11(12):e655. doi: 10.1002/ctm2.655 (PMC8694501; doi:10.1002/ctm2.655)
Supplement: Supplementary file 1 — Supporting Information [file CTM2-11-e655-s001.docx]

**INTEGRATED MULTI-OMICS DATA REVEALS THE MOLECULAR SUBTYPES AND GUIDES THE ANDROGEN RECEPTOR SIGNALING INHIBITOR TREATMENT OF PROSTATE CANCER**

**SUPPLEMENTARY MATERIALS AND METHODS …………………..…. Page 2**

**SUPPLEMENTARY TABLES S1-6 …………………………………….……. Page 7**

**SUPPLEMENTARY FIGURES S1-9 ………………………………….……... Page 12**

**Methods and materials**

**Data collection and preparation**

Seven prostate cancer (PCa) cohorts were enrolled in the current study. TCGA-PARD cohort contained 495 PCa patients was used as the training cohort, due to the perfect record of multi-omics data and clinical information. For GEO cohorts, GSE116918[1], GSE70770[2], MSKCC[3] and GSE54460[4] were enrolled to reproduce and validation new findings in TCGA-PRAD training cohort. Abida cohort containing the gene matrix and response status to androgen deprivation (ADT) therapy was also employed to evaluate the sensitive to ADT[5]. AHMU-PC cohort, with 69 PCa patients collected from our own institute and sequenced as described in the previous study[6] was also enrolled. The detailed clinical information of these cohorts are listed in **Table S1 and Table S2**.

We used the TCGA-PRAD cohort as the training cohort, and data on mRNA expression, lncRNA expression, miRNA expression, DNA methylation and somatic mutations were enrolled to identify the different subtypes linked with relapse status and time. The row counts of mRNA and lncRNA were collected through the “*TCGAbiolinks*” [7]. For the gene expression factors, the gene symbols were transformed after mapping the Ensembl IDs by the GENCODE27 annotation. Ensembl IDs recognized as the lncRNAs through Vega (<http://vega.archive.ensembl.org/>) were summarized as the lncRNA input file. The raw counts of miRNAs were obtained from the UCSC Xena and translated to the mature form IDs recorded in miRbase version 21.0 via the R package “*miRNAmeConverter*”. The raw read counts were transferred into transcripts per kilobase million (TPM) values. The β values of DNA methylation were captured from the Illumina DNA methylation 450 platform from the UCSC Xena website. Furthermore, we downloaded the gene mutation file, clinicopathological features and recurrence-free survival (RFS) from cBioPortal (<https://www.cbioportal.org/>). After merging the available samples in all omics and clinical features, a total of 486 PCa patients were left for subsequent analysis. Four PCa patient cohorts recorded available clinicopathological information, and RFS data were also enrolled as the external validation cohort, including the GSE116918, GSE70770, MSKCC and GSE54460 cohorts. The gene symbol for each cohort was transferred from the probe ID according to the corresponding annotation file from each platform. For one gene symbol that was mapped by multiple probes, the mean value was calculated as the expression level.

**Integration of multi-omics and recognition of subtypes**

We performed the recognition of subtypes of PCa patients based on mRNA expression, lncRNA expression, miRNA expression, DNA methylation and somatic mutations data. The TPM expression data of mRNA, lncRNA and miRNA were first transformed by log2 calculation. As to DNA methylation, we only concerned the probes around the promoter of genes. For the gene mutation matrix, we considered the gene as having mutant status if it contained any of the following nonsynonymous variations: frameshift deletion/insertion, in-frame deletion/insertion, missense/nonsense/nonstop mutation, splice site or translation start site mutation. To identify the subtypes that could mostly separate the different clinical outcomes for PCa patients, we selected the top factors most highly related to RFS (*P* value less than 0.01 from Cox regression analysis), including 1526 mRNA, 242 lncRNA, 30 miRNA, and 1073 DNA CpG methylation sites, and 23 mutant genes had a mutation rate higher than 0.3. Subsequently, we selected the number of subtypes based on the prediction results of the clustering prediction index (CPI) [8] and Gaps-statistics [9]. Then, we performed clustering via 10 state-of-the-art multi-omics clustering algorithms, including iClusterBayes, moCluster, CIMLR, IntNMF, ConsensusClustering, COCA, NEMO, PINSPlus, SNF, and LRA. The final subtype clustering combines the results of the 10 algorithms mentioned above to make the clustering more robust.

**Specific activated signaling pathways among subtypes**

To reveal the specific characteristics of each subtype, we compared the distribution of oncogenic pathways, metabolic pathways and infiltration of immunocytes in the newly recognized subtypes. The enrichment score of single-sample gene set enrichment analysis (ssGSEA) for all concerned pathways was also calculated[10]. For the oncogenic pathways, we used the 50 tumor-associated gene sets from HALLMARK[11]. For the metabolic pathways, we enrolled a total of 113 gene sets obtained from a published article[12]. We also assessed the infiltration rate of immunocytes with the CIBERSORT algorithm[13], and the results of immunocytes with an average infiltration value of 0.01 were discarded. The functional proteomic data of 218 proteins were downloaded from The Cancer Proteome Atlas (TCPA) project, which used a reversed-phase protein array to detect the level of proteins combined with antibodies[14]. To compare the mRNA or protein levels between different subtypes, the average level of each subtype was first determined, and then the level of each gene or protein among the subtypes was transferred to the normal distribution to make all of the results comparable.

**Transcriptional regulatory networks among three PMOC subtypes**

We assessed the activation of tumorigenesis pivotal transcriptional regulatory networks (regulons) composed of the transcription factor and its downstream target genes. The analysis of regulons was conducted by the package “*RTN*”, as previously reported [15]. Moreover, we evaluated the impact of 71 chromatin remodeling-associated genes, which are regarded as potential gene regulators [16]. We analyzed the mutual information and Spearman’s correlation to evaluate the potential associations among regulators and downstream target genes through the gene expression matrix, and associations identified by false discovery rate (FDR)-adjusted p-values higher than 0.00001 evaluated by permutation analysis were discarded. In addition, shaky links were eliminated via the 1,000 re-samplings bootstrap analysis, as well as the removing of weak associations between regulators and target genes via the filtering of data processing inequality. GSEA was used to compute the regulon activity scores, and visualized by heatmap.

**Evaluation of AR activity and response ADT therapy**

Two previously reported AR activity signatures were collected, and the activation score was also calculated by GSEA. Farzana et al.[17] reported the AR Activity (AR-A) score, which is analyzed by the weighted linear summarize of KLK3, KLK2, FKBP5, STEAP1, STEAP2, PPAP2A, RAB3B, ACSL3, NKX3-1. Haley et al. refined the AR activation signature with 27 genes that show robust activation or inhibition of expression upon androgen stimulation, including KLK3, TMPRSS2, NKX3-1, KLK2, GNMT, PMEPA1, MPHOSPH9, ZBTB10, EAF2, CENPN, C1orf116, ACSL3, PTGER4, ABCC4, NNMT, ADAM7, FKBP5, ELL2, MED28, HERC3, MAF, TNK1, GLRA2, MAPRE2, PIP4K2B, MAN1A1, and CD200. In the current study, we used the genes from AR-A score and AR activation signature to generate the corresponding score reflect the activated status of AR signaling by ssGSEA. A clinical cohort containing the gene matrix and response status to ADT therapy was also employed to evaluate the diverse response to ADT among three PMOC subtypes[5]. The estimated IC_50_ of bicalutamide, an AR signaling inhibitor (ARSI), in patients was calculated by the R package “*pRRophetic*” after comparison with the recorded response IC_50_ value of the drug in urinary tumor cell lines by the10-fold cross-validation via ridge regression [18].

**Characteristics of genetic alterations among subtypes**

The diversity distribution of mutant genes among subtypes was compared by Chi-square test and displayed with a triangle plot, in which the coordinates of each point represent the mutation frequency of the gene, and the closer a point is to a corner of the triangle, the more mutation is present in the subtype. The size of the point is linked with the total mutation frequency of the gene. The mRNA expression among wild-type and mutant APC samples was compared by the Wilcoxon test, and the clinical outcome virialized by cumulative recurrent events plot.

**Statistics**

We performed all the statistical and bioinformatical analysis via the R 4.0.2. Mann-Whitney test and Student t-test employed for the comparison of continuous data. Kaplan-Meier plot, log-rank test, and Cox regression analysis were applied to reveal the different RFS outcome, hazard ratio (HR) with a 95% confidence interval (95% CI). The biological differences in different LumB subtypes identified were evaluated by GSEA through the R package “*clusterProfiler*” [19]. Specifically, GSEA was performed with the limma analysis generated log2FoldChange value sorted gene list. For the external validation cohort, the top 100 most differentially expressed genes sorted by log2FoldChange for each PMOC were chosen as the biomarkers, these biomarkers should pass the significance threshold (e.g., nominal P value < 0.05 and adjusted P value < 0.05) and must not overlap with any biomarkers identified for other subtypes, then the nearest template prediction (NTP) analysis was conducted to reveal the subtypes with the 300 specific marker genes generated from TCGA-PRAD [20]. We conducted most of the analyses via the multi-omics integration analysis R package “MOVICS”[21], which is available in the Github (<https://github.com/xlucpu/MOVICS>). For all the results, *P* < 0.05 was considered as the significant different.

**References**

1. Jain S, Lyons CA, Walker SM, McQuaid S, Hynes SO, Mitchell DM, et al. Validation of a Metastatic Assay using biopsies to improve risk stratification in patients with prostate cancer treated with radical radiation therapy. Ann Oncol. 2018; 29: 215-22.

2. Ross-Adams H, Lamb AD, Dunning MJ, Halim S, Lindberg J, Massie CM, et al. Integration of copy number and transcriptomics provides risk stratification in prostate cancer: A discovery and validation cohort study. EBioMedicine. 2015; 2: 1133-44.

3. Taylor BS, Schultz N, Hieronymus H, Gopalan A, Xiao Y, Carver BS, et al. Integrative genomic profiling of human prostate cancer. Cancer Cell. 2010; 18: 11-22.

4. Long Q, Xu J, Osunkoya AO, Sannigrahi S, Johnson BA, Zhou W, et al. Global transcriptome analysis of formalin-fixed prostate cancer specimens identifies biomarkers of disease recurrence. Cancer Res. 2014; 74: 3228-37.

5. Abida W, Cyrta J, Heller G, Prandi D, Armenia J, Coleman I, et al. Genomic correlates of clinical outcome in advanced prostate cancer. Proc Natl Acad Sci U S A. 2019; 116: 11428-36.

6. Meng J, Zhou Y, Lu X, Bian Z, Chen Y, Zhou J, et al. Immune response drives outcomes in prostate cancer: implications for immunotherapy. Mol Oncol. 2021; 15: 1358-75.

7. Colaprico A, Silva TC, Olsen C, Garofano L, Cava C, Garolini D, et al. TCGAbiolinks: an R/Bioconductor package for integrative analysis of TCGA data. Nucleic acids research. 2015; 44: e71-e.

8. Chalise P, Fridley BL. Integrative clustering of multi-level ‘omic data based on non-negative matrix factorization algorithm. PloS one. 2017; 12: e0176278.

9. Hastie T, Tibshirani R, Walther G. Estimating the number of data clusters via the Gap statistic. J Roy Stat Soc B. 2001; 63: 411-23.

10. Dreyer SB, Upstill-Goddard R, Paulus-Hock V, Paris C, Lampraki E-M, Dray E, et al. Targeting DNA Damage Response and Replication Stress in Pancreatic Cancer. Gastroenterology. 2021; 160: 362-77.e13.

11. Liberzon A, Birger C, Thorvaldsdottir H, Ghandi M, Mesirov JP, Tamayo P. The Molecular Signatures Database (MSigDB) hallmark gene set collection. Cell Syst. 2015; 1: 417-25.

12. Rosario SR, Long MD, Affronti HC, Rowsam AM, Eng KH, Smiraglia DJ. Pan-cancer analysis of transcriptional metabolic dysregulation using The Cancer Genome Atlas. Nat Commun. 2018; 9: 5330.

13. Newman AM, Liu CL, Green MR, Gentles AJ, Feng W, Xu Y, et al. Robust enumeration of cell subsets from tissue expression profiles. Nature methods. 2015; 12: 453.

14. Li J, Lu Y, Akbani R, Ju Z, Roebuck PL, Liu W, et al. TCPA: a resource for cancer functional proteomics data. Nat Methods. 2013; 10: 1046-7.

15. Robertson AG, Kim J, Alahmadie H, Bellmunt J, Guo G, Cherniack AD, et al. Comprehensive Molecular Characterization of Muscle-Invasive Bladder Cancer. Cell. 2017.

16. Audia JE, Campbell RM. Histone Modifications and Cancer. Cold Spring Harb Perspect Biol. 2016; 8: a019521-a.

17. Faisal FA, Sundi D, Tosoian JJ, Choeurng V, Alshalalfa M, Ross AE, et al. Racial Variations in Prostate Cancer Molecular Subtypes and Androgen Receptor Signaling Reflect Anatomic Tumor Location. Eur Urol. 2016; 70: 14-7.

18. Geeleher P, Cox NJ, Huang RS. Clinical drug response can be predicted using baseline gene expression levels and in vitro drug sensitivity in cell lines. Genome biology. 2014; 15: R47.

19. Yu G, Wang L-G, Han Y, He Q-Y. clusterProfiler: an R package for comparing biological themes among gene clusters. Omics: a journal of integrative biology. 2012; 16: 284-7.

20. Hoshida Y. Nearest template prediction: a single-sample-based flexible class prediction with confidence assessment. PloS one. 2010; 5: e15543.

21. Lu X, Meng J, Zhou Y, Jiang L, Yan F. MOVICS: an R package for multi-omics integration and visualization in cancer subtyping. Bioinformatics. 2020.

**SUPPLEMENTARY TABLES**

**Table S1. Clinicopathological features of five enrolled cohorts.**

|  | **TCGA-PRAD** | **GSE116918** | **GSE70770** | **MSKCC** | **GSE54460** | **AHMU-PC** |
| --- | --- | --- | --- | --- | --- | --- |
| **Patients, number** | **495** | **248** | **203** | **140** | **106** | **69** |
| **Age, years old** | 61.03 ±  6.84 | 60.46 ± 6.59 | 67.35 ± 6.36 | 58.13 ± 6.97 | 61.11 ± 6.68 | 69.16 ± 8.43 |
| **Gleason^*^** |  |  |  |  |  |  |
| **6** | 45 | 42 | 35 | 41 | 10 | 16 |
| **7** | 246 | 99 | 140 | 76 | 80 | 21 |
| **8** | 63 | 52 | 13 | 11 | 10 | 13 |
| **9** | 137 | 54 | 10 | 10 | 5 | 14 |
| **10** | 4 | 1 | 1 | - | - | 2 |
| **PSA^#^, ng/dl** |  |  |  |  |  |  |
| **≤10** | 422 | 50 | 145 | 114 | 72 | 29 |
| **>10** | 16 | 198 | 55 | 24 | 31 | 39 |
| **Stage^†^** |  |  |  |  |  |  |
| **T1** | - | 51 | - | - | 14 | - |
| **T2** | 187 | 76 | 81 | 86 | 73 | 55 |
| **T3** | 291 | 92 | 118 | 47 | 17 | 12 |
| **T4** | 10 | 4 | 1 | 7 | 1 | 2 |

^*^ Lack of Gleason score: 4 in GSE70770, 2 in MSKCC, 1 in GSE54460, 3 in AHMU-PC;

^#^ Lack of PSA value: 57 in TCGA-PRAD, 3 in GSE70770, 2 in MSKCC, 3 in GSE54460, 1 in AHMU-PC;

^†^ Lack of T Stage value: 7 in TCGA-PRAD, 25 in GSE116918, 3 in GSE70770, 1 in GSE54460.

**Table S2. Basic information of the enrolled five prostate cancer cohorts.**

| **Cohorts** | **Archive** | **Platform** | **Platform** | **Data Type** | **Sample Size** |
| --- | --- | --- | --- | --- | --- |
| **TCGA-BLCA** | TCGA | Illumina HiSeq 2000 RNA Sequencing | - | RNA-Seq | 495 |
| **GSE116918** | GEO | Almac Diagnostics Prostate Disease Specific Array (DSA) | GPL25318 | Microarray | 248 |
| **GSE70770** | GEO | Illumina HumanHT-12 V4.0 expression beadchip | GPL10558 | Microarray | 203 |
| **GSE54460** | GEO | Illumina HiSeq 2000 | GPL11154 | Microarray | 140 |
| **MSKCC** | GEO | Affymetrix Human Exon 1.0 ST Array | GPL5188  GPL10264 | RNA-Seq | 106 |
| **AHMU-PC** | Our institute | Illumina Pe150 sequencing | - | High throughput sequencing | 69 |

Table S3. The distribution of the clinical features among three prostate cancer molecular subtypes

| Prameters | | PMOC1 | PMOC2 | PMOC3 | *P* |
| --- | --- | --- | --- | --- | --- |
| **Patients (n)** |  | 209 | 123 | 154 |  |
| **Recurrent status (%)**^†^ | Non-recurrent | 171 (81.8) | 77 (62.6) | 147 (95.5) | <0.001** |
|  | Recurrent | 38 (18.2) | 46 (37.4) | 7 (4.5) |  |
| **Recurrent-free survival time, months**  **(median [IQR]) ^$^** |  | 23.51 [12.98, 38.00] | 20.89 [7.08, 34.21] | 36.41 [23.15, 49.66] | <0.001** |
| **Age, years, (mean± SD)** ^#^ |  | 60.94 (6.67) | 62.46 (6.70) | 59.99 (7.09) | 0.012* |
| **Gleason, n (%)**^‡^ | 6 | 25 (12.0) | 1 (0.8) | 19 (12.3) | <0.001** |
|  | 7 | 111 (53.1) | 22 (17.9) | 108 (70.1) |  |
|  | 8 | 25 (12.0) | 24 (19.5) | 12 (7.8) |  |
|  | 9 | 48 (23.0) | 74 (60.2) | 14 (9.1) |  |
|  | 10 | 0 (0.0) | 2 (1.6) | 1 (0.6) |  |
| **PSA, n (%)**^†^ | <=4 | 178 (85.2) | 92 (74.8) | 134 (87.0) | 0.050 |
|  | >4 | 8 (3.8) | 11 (8.9) | 8 (5.2) |  |
|  | unknow | 23 (11.0) | 20 (16.3) | 12 (7.8) |  |
| **T stage, n (%)**^‡^ | T2 | 93 (44.5) | 16 (13.0) | 75 (48.7) | <0.001** |
|  | T3 | 112 (53.6) | 101 (82.1) | 73 (47.4) |  |
|  | T4 | 1 (0.5) | 4 (3.3) | 4 (2.6) |  |
|  | unknow | 3 (1.4) | 2 (1.6) | 2 (1.3) |  |

*, P<0.05, **, P<0.01; †, Chi-square test; ‡, Fisher extract test; #, One-way ANOVA test; $, Kruskal- Aallis test; †，

**Table S4. Template genes for three subtypes**

| **PMOC1-Inflammatory** | **PMOC2-Activated** | **PMOC3-Balance** |
| --- | --- | --- |
| KRT15 | UBE2C | OR51E2 |
| WFDC2 | RGS11 | ACPP |
| PCP4 | SCHLAP1 | FMOD |
| DES | COMP | ST6GAL1 |
| TPM2 | CDC20 | EHF |
| CDC42EP5 | TPX2 | PCA3 |
| VSIG2 | MYBL2 | ENTPD5 |
| MIR205HG | TK1 | ALOX15B |
| MYL9 | TOP2A | DPP4 |
| CNN1 | AC131902.1 | SLC2A12 |
| KRT17 | SSTR1 | KIAA1324 |
| PAGE4 | BIRC5 | LPAR3 |
| PTGDS | NUSAP1 | PRUNE2 |
| KRT5 | CDK1 | HMGPMOC2 |
| COX7A1 | PDIA2 | AC113191.1 |
| GSTP1 | PTTG1 | AC005831.1 |
| ID1 | CRISP3 | CYP1B1 |
| RARRES2 | AC131902.3 | SPOCK1 |
| AC020916.1 | UBE2T | SLC26A2 |
| TAGLN | B4GALNT4 | SERPINA11 |
| S100A6 | BMP6 | FAM129A |
| COL7A1 | AMH | ZNF770 |
| KRT14 | KLK14 | SEL1L |
| AC053503.4 | DLX1 | GCNT1 |
| MT1M | ASF1B | AC022034.2 |
| SNCG | AC092535.4 | PLA2G2A |
| CFD | ARHGDIG | RDH11 |
| TCEAL2 | COL10A1 | PCYOX1 |
| PPP1R14A | AC069228.1 | DNAJC3 |
| KRT13 | KIFC1 | SLC30A4 |
| MT1A | CPNE7 | ARG2 |
| KRT7 | HES6 | SERINC5 |
| CEBPD | GPC5-AS1 | IQGAP2 |
| LAMB3 | RRM2 | STEAP4 |
| IGFBP6 | LMNB1 | PGC |
| C11ORF96 | AL162413.1 | ITPR1 |
| FABP3 | CENPU | RAB3B |
| SCGB3A1 | ZNF467 | PTPRN2 |
| AZGP1 | E2F1 | RAB27B |
| SMTN | AC093001.1 | NCAPD3 |
| AC025259.3 | PPFIA2 | GNE |
| 7SK | CDCA5 | CPD |
| NBL1 | TACC3 | GHR |
| SLC14A1 | CCDC78 | PCDHGC3 |
| DEFB1 | STMN1 | CD302 |
| S100A14 | KIF4A | BHLHA15 |
| MT1X | HJURP | SCD |
| RPL29P14 | KIF20A | LIFR |
| PDLIM7 | CKS2 | IGF2R |
| MT1E | TMEM132A | SLC4A4 |
| TGM4 | NBEAP1 | ABAT |
| PHYHD1 | TROAP | PARM1 |
| LGALS1 | FABP5 | ATP8B1 |
| LINC00844 | FOXM1 | ACER2 |
| S100A2 | MMP11 | SEMA3C |
| SNHG18 | AC239868.2 | FZD5 |
| CPXM1 | CTBP1-AS | AFF3 |
| MT2A | PRC1 | ITPR2 |
| TRIM29 | PTK6 | USP34 |
| GRASP | SPAG5 | LPCAT3 |
| LIMS2 | AC023310.4 | ADAM9 |
| NTF4 | DEGS1 | FRK |
| PDLIM4 | CENPA | CPE |
| ACKR1 | CCNA2 | RBM47 |
| MRGPRF | MNX1-AS1 | CDH1 |
| GSTM2 | NEK2 | AC005336.2 |
| CLU | MELK | PJA2 |
| VAMP5 | SNHG4 | GOLM1 |
| CCL2 | CENPF | DSG2 |
| SELENOM | CST2 | PCAT14 |
| TRIP6 | CDKN3 | VCL |
| UBXN10-AS1 | AL139246.5 | UGGT1 |
| S100A4 | AURKB | GALNT7 |
| CXCL2 | CDT1 | KCND3 |
| S100A16 | MKI67 | SEL1L3 |
| TIMM8AP1 | AC009478.1 | AL049779.2 |
| SYCE1L | RAB26 | FAT1 |
| DLK2 | GRIN3A | SLC12A2 |
| TNS4 | IQANK1 | AL035413.2 |
| TGFB1I1 | PBK | AL354984.2 |
| CAVIN3 | CCNB1 | DENND4C |
| ADAM33 | ISG15 | AC015922.3 |
| MTND4P12 | U73166.1 | ABCC4 |
| FAM46B | MIR4292 | CACNA2D1 |
| CRYAB | RPL39P40 | FKBP5 |
| CLDN5 | SPC24 | FAM13C |
| MEG3 | PLK1 | SEC24C |
| SCGB1A1 | B3GNT6 | APP |
| ANPEP | CDCA8 | ABHD2 |
| CCK | AURKA | CNTNAP2 |
| HSPB1 | PRKAG2-AS1 | GP2 |
| IFI27L2 | PTPRT | BRINP3 |
| SGCA | MEX3A | DSC2 |
| ORM2 | HAGHL | LMAN1 |
| SAA1 | FAM111B | AC024075.1 |
| KRT23 | ESM1 | HIPK2 |
| MIRLET7D | NCAPH | ZNF106 |
| LCAT | PKMYT1 | ITGB1 |
| EPHB6 | AP002498.1 | GUCY1A3 |
| CXCL1 | NKAIN1 | F3 |

**Table S5. The difference distribution of gene mutations in three phenotypes.**

| **Gene** | **Total** | **PMOC1** | **PMOC2** | **PMOC3** | ***P* value** |
| --- | --- | --- | --- | --- | --- |
| TP53 | 56 (11.5%) | 18 (8.6%) | 29 (23.6%) | 9 (5.8%) | 1.81E-05 |
| ABCA13 | 12 (2.5%) | 5 (2.4%) | 7 (5.7%) | 0 (0.0%) | 4.62E-03 |
| RYR1 | 15 (3.1%) | 8 (3.8%) | 7 (5.7%) | 0 (0.0%) | 5.36E-03 |
| APC | 10 (2.1%) | 2 (1.0%) | 7 (5.7%) | 1 (0.6%) | 9.66E-03 |
| PTEN | 18 (3.7%) | 2 (1.0%) | 7 (5.7%) | 9 (5.8%) | 1.07E-02 |
| SPOP | 55 (11.3%) | 20 (9.6%) | 23 (18.7%) | 12 (7.8%) | 1.38E-02 |
| CFH | 10 (2.1%) | 1 (0.5%) | 6 (4.9%) | 3 (1.9%) | 1.58E-02 |
| XIRP2 | 10 (2.1%) | 1 (0.5%) | 6 (4.9%) | 3 (1.9%) | 1.58E-02 |
| DCHS2 | 12 (2.5%) | 4 (1.9%) | 7 (5.7%) | 1 (0.6%) | 3.19E-02 |
| STAB2 | 10 (2.1%) | 7 (3.3%) | 3 (2.4%) | 0 (0.0%) | 3.87E-02 |
| MUC17 | 15 (3.1%) | 3 (1.4%) | 8 (6.5%) | 4 (2.6%) | 4.68E-02 |
| SPTA1 | 23 (4.7%) | 6 (2.9%) | 11 (8.9%) | 6 (3.9%) | 4.90E-02 |
| USH2A | 15 (3.1%) | 5 (2.4%) | 8 (6.5%) | 2 (1.3%) | 4.99E-02 |

**SUPPLEMENTARY FIGURES**

**
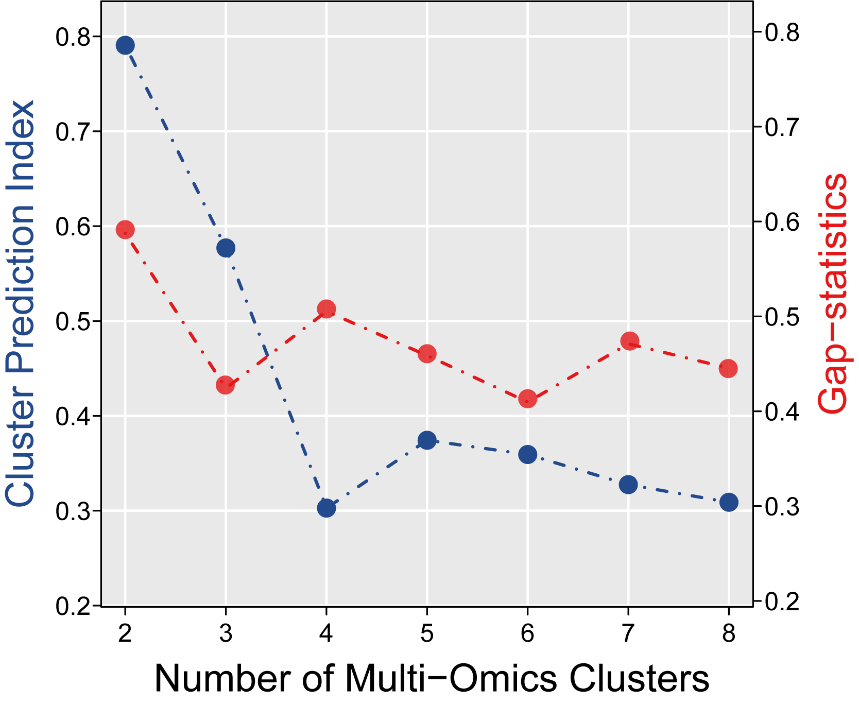
**

**Figure S1. Speculation regarding the appropriate clustering number by CPI and Gap-statistics analysis.**

**
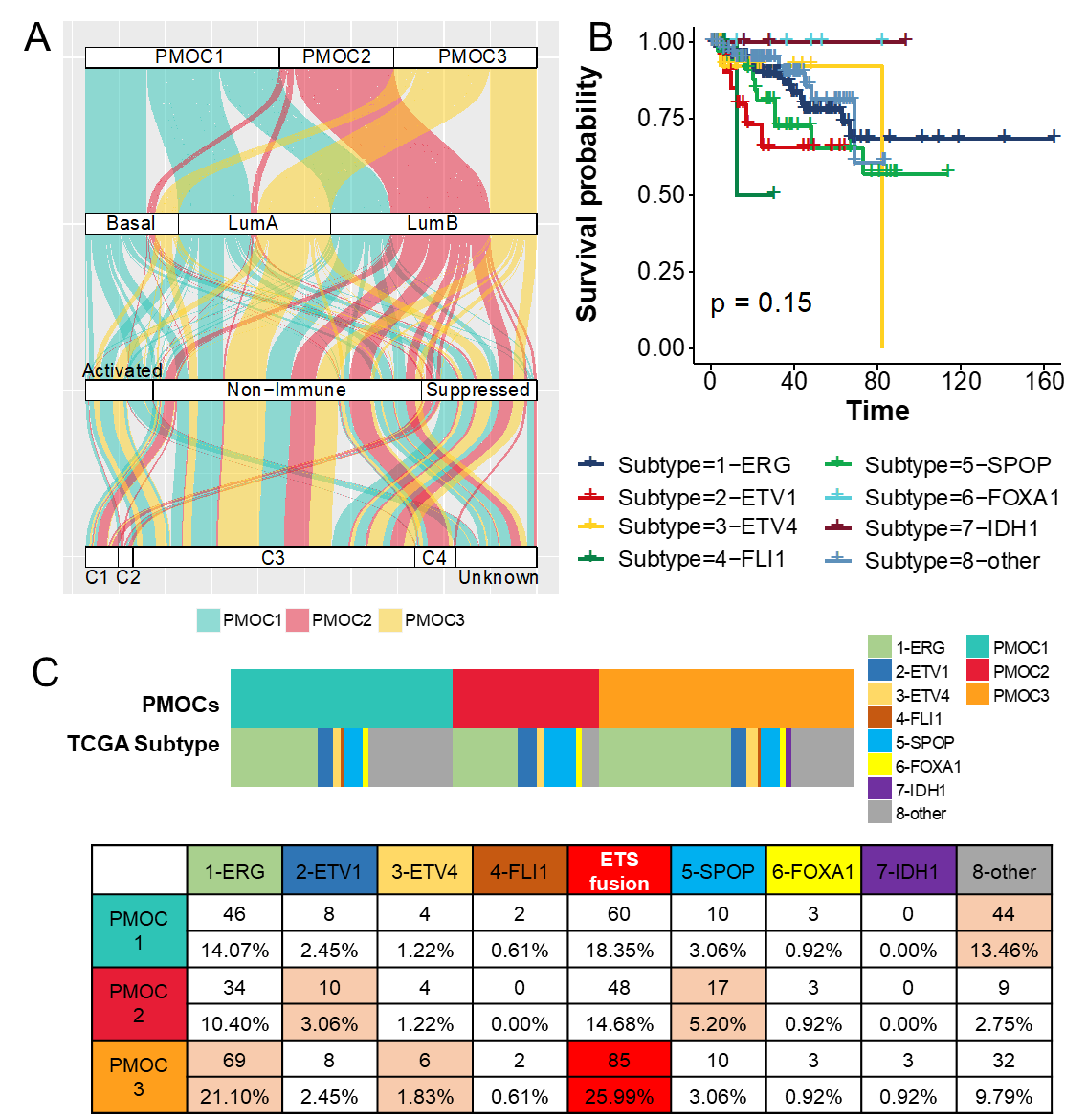
**

**Figure S2. Comparison of PMOCs with proposed PCa subtypes.** (A) Overlap between PMOCs, PAM50, immune activation subtypes and six immune molecular features; (B) Diverse recurrence-free survival outcome among TCGA subtypes, log-rank test; (C) Comparison between PMOCs and TCGA subtypes.

**
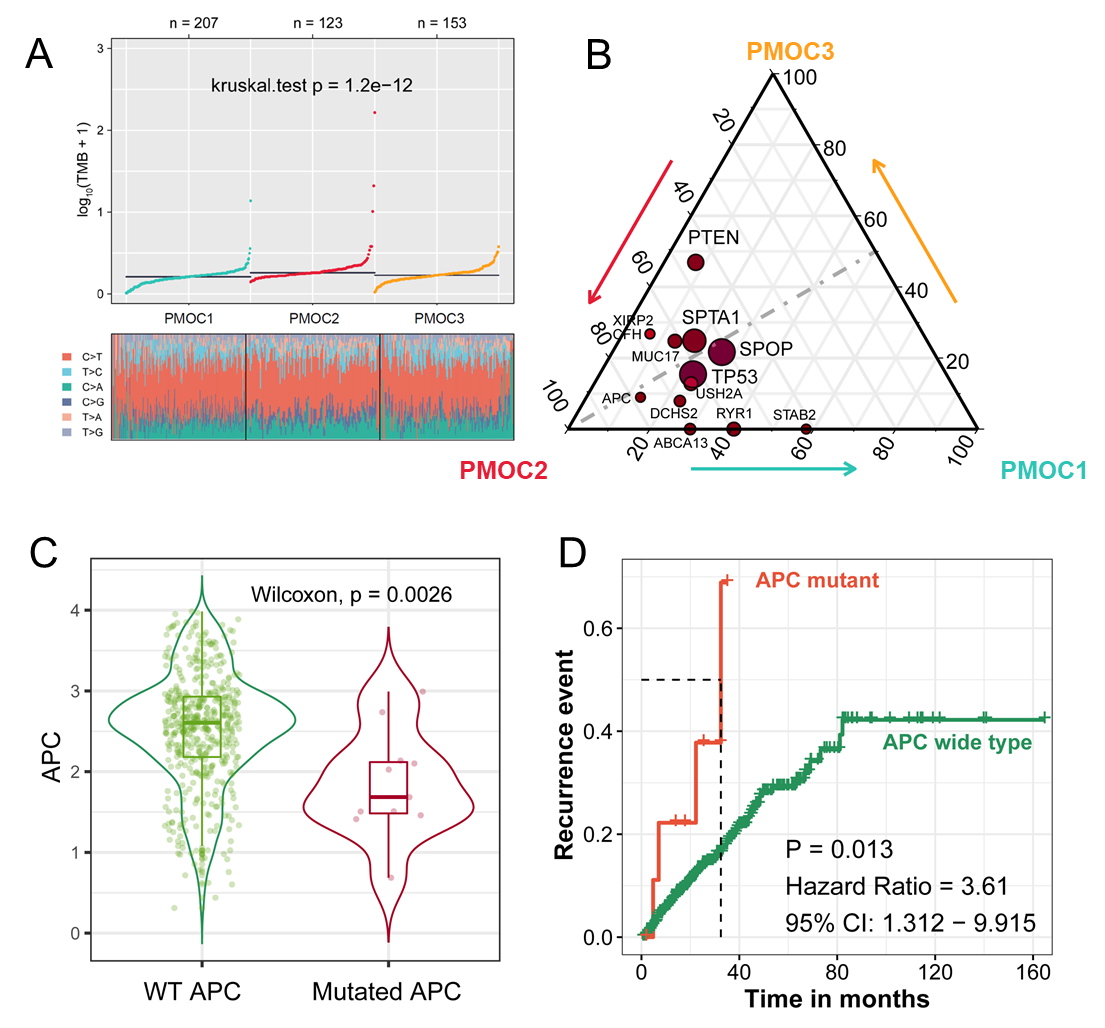
**

**Figure S3. Gene mutation landscape among three PMOCs.** (A) TMB difference in three PMOCS, Kruskal-Wallis test; (B) Triangle plot showing the differential mutation frequency of tumor-driven mutant genes; (C) Mutated APC caused the downregulation of self mRNA expression, Wilcoxon test; (D) Mutated APC resulted in the greater accumulation of recurrence events, log-rank test.


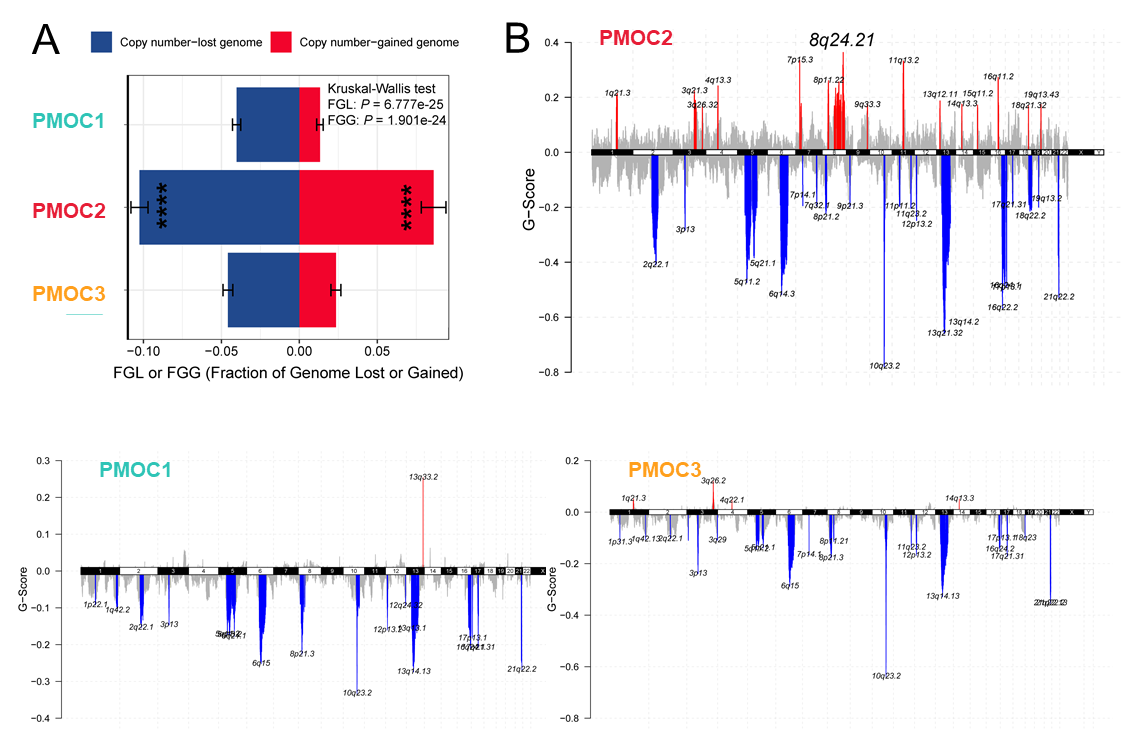


**Figure S4. Copy number alteration landscape among three PMOCs.** (A) Disparities in copy numbers gained and lost in PMOCs, Kruskal-Wallis test; (B) Plot of G scores calculated by GISTIC 2.0 for amplified (red) or deleted (blue) in the PMOCs.


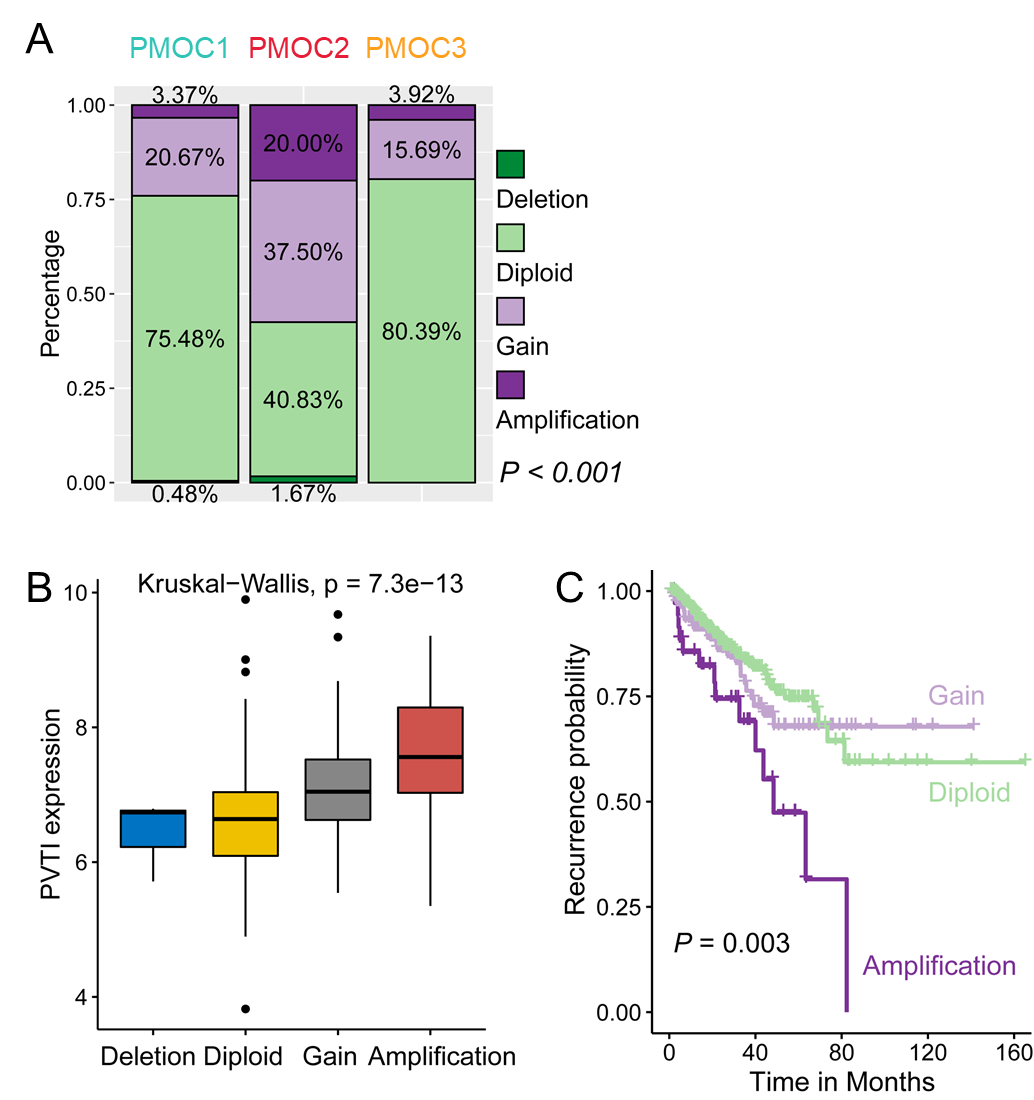


**Figure S5. PVT1 amplification status in 8q24.21 region.** (A) Copy number alteration of PVT1 in three PMOCs, Fisher’s extract test; (B) Correlation of copy number alteration with mRNA expression of PVTI; (C) Amplification of PVTI indicated the worst clinical outcome, log-rank test.

**
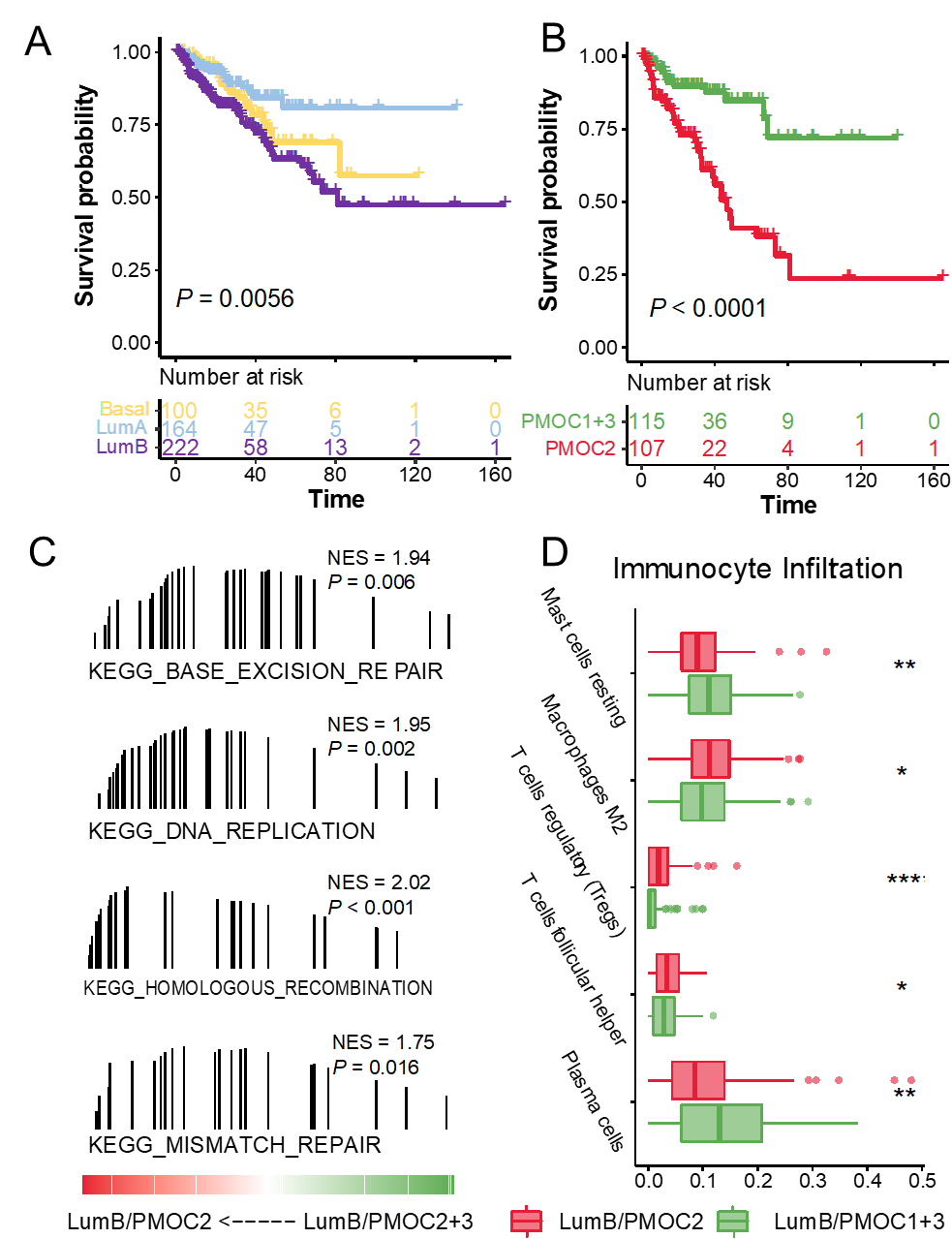
**

**Figure S6. PMOC subtypes separated the PCa luminal B molecular subtype with diverse recurrence outcomes.** (A) Differential clinical outcomes in the basal, LumA and LumB subtypes, log-rank test; (B) Differential clinical outcomes in the LumB/PMOC1+3 and LumB/PCS2 subtypes, log-rank test; (C) DNA replication and repair pathways activated in LumB/PMOC2 compared with LumB/PMOC1+3, GSEA analysis; (D) Differential infiltration of immunocytes in two separate LumB subtypes, Wilcoxon test.


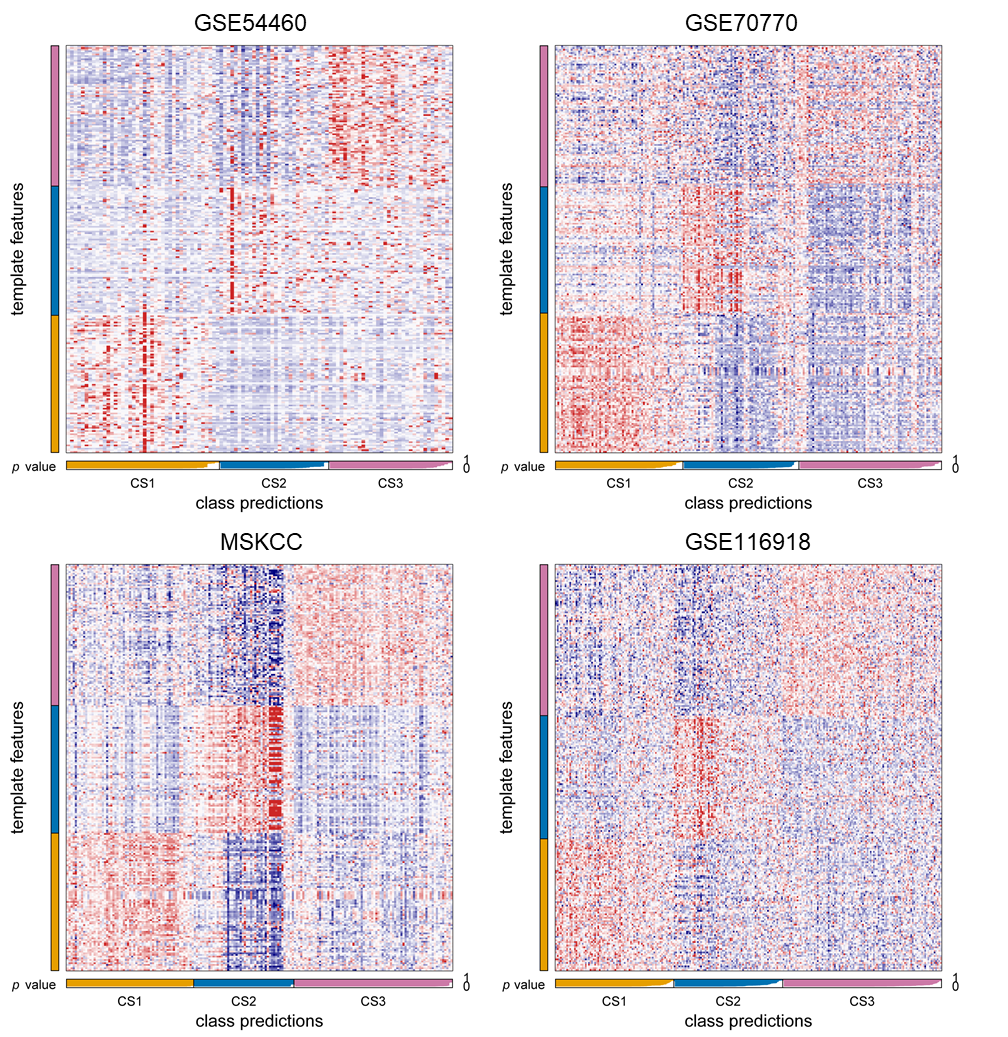


**Figure S7. Recognizing the three subtypes by the template genes in four external validation cohorts.**
